# Abdominal Aortic Screening Is a Priority for Health in Smoker Males: A Study on Central Italian Population

**DOI:** 10.3390/ijerph19010591

**Published:** 2022-01-05

**Authors:** Emma Altobelli, Filippo Gianfelice, Paolo Matteo Angeletti, Reimondo Petrocelli

**Affiliations:** 1 Department of Life, Public Health and Environmental Sciences, University of L’Aquila, 67100 L’Aquila, Italy; paolomatteoangeletti@gmail.com; 2 Vascular Surgery, G. Mazzini di Teramo Hospital, Local Health Unit, 64100 Teramo, Italy; filippo.gianfelice@virgilio.it; 3 Rianimazione e TIPO Cardiochirurgica, Ospedale G. Mazzini, Local Health Unit, 64100 Teramo, Italy; 4 S. Timoteo Hospital, ASREM, 86100 Campobasso, Italy; r.petrocelli@libero.it

**Keywords:** abdominal aortic aneurysm, prevalence, screening, risk factors and cost-effectiveness

## Abstract

Abdominal aortic aneurysm (AAA) is a major public health problem. In the last decade, in some European countries, abdominal aortic screening (AAS) is emerging as a potential prevention for the rupture of AAA. The goals of our study were to estimate AAA prevalence and risk factors in males and females in a central Italian population, also defining the cost-effectiveness of AAS programs. A pilot study screening was conducted between 1 January 2015 and 31 December 2019 in the municipality of Teramo (Abruzzo Region, Italy) in a group of men and women, ranging from the age of 65 to 79, who were not previously operated on for AAA. The ultrasound was performed by means of Acuson sequoia 512 Simens with a Convex probe. The anterior posterior of the infra-renal aorta was evaluated. The odds ratio values (ORs) were used to evaluate the risk of AAA, and the following determinants were taken into consideration: gender, smoke use, hypertension, and ischemic heart disease. We also estimated the direct costs coming from aneurysmectomy (surgical repair or endovascular aneurysms repair—EVAR). A total of 62 AAA (2.7%, mean age 73.8 ± 4.0) were diagnosed, of which 57 were in men (3.7%, mean age 73.6 ± 4.0) and 5 were in women (0.7%, mean age 74.3 ± 4.1). Male gender and smoke use are more important risk factors for AAA ≥ 3 cm, respectively: OR = 5.94 (2.37–14.99, *p* < 0.001) and OR = 5.21 (2.63–10.30, *p* < 0.000). A significant increase in OR was noted for AAA ≥ 3 cm and cardiac arrhythmia and ischemic heart disease, respectively: OR = 2.81 (1.53–5.15, *p* < 0.000) and OR = 2.76 (1.40–5.43, *p* = 0.006). Regarding the cost analysis, it appears that screening has contributed to the reduction in costs related to urgency. In fact, the synthetic indicator given by the ratio between the DRGs (disease related group) relating to the emergency and those of the elective activity went from 1.69 in the year prior to the activation of the screening to a median of 0.39 for the five-year period of activation of the screening. It is important to underline that the results of our work confirm that the screening activated in our territory has led to a reduction in the expenditure for AAA emergency interventions, having increased the planned interventions. This must be a warning for local stakeholders, especially in the post-pandemic period, in order to strengthen prevention.

## 1. Introduction

Abdominal aortic aneurysm (AAA) is a major public health problem. It consists of a dilation of the AAA ≥ 3 cm [1] that, in 85% of cases, occurs in the infra-renal segment of the abdominal aorta [2].

AAA prevalence rates have decreased in the last decades from 7.2 to 3.9 [3,4]; currently, AAA has a range between 1.2% and 3.3% [5,6].

Recent data show that the risk of rupture is 3.5% for a diameter between 5.5 and 6 cm, 4.1% between 6.1 and 7 cm, and 6.3% if ≥7 cm [7]. The real danger of the AAA consists in the fact that it is often asymptomatic until its rupture, causing mortality in 60–80% of cases [8]. In fact, an important proportion of patients die before they arrive in the operating room [9]. On the other hand, if the surgical correction is made with an intervention schedule, there is an important reduction in mortality of between 2 and 4% [10]. The ultrasound is diagnostic if conducted by expert personnel, with a sensitivity of 95% and a specificity of 100% [9]. The Preventive Service Task Force recommends AAA screening by ultrasonography in men aged 65 to 75 years who have smoked, but not among non-smokers [11]. In fact, the AAA prevalence is four times higher in smokers than in non-smokers [12]. Ahmed et al. reported that patients with AAA were often smoker males [13]. Regarding cardiovascular diseases, smoking was found to be the major risk factor associated with AAA [14].

It is important to underline that the growth rate of aneurysms is higher in smokers [15]. Among younger patients, a higher frequency was found in subjects with high cardiovascular risk [16].

However, although important systematic reviews and meta-analysis effectiveness of this screening has been demonstrated [17,18,19], it has not been implemented in all developed countries yet, perhaps due to a lack of dedicated economic resources. AAA screening programs have been set up in the UK, Sweden, and the USA [20]. These programs use ultrasonography to screen AAA with a dilation of the AAA ≥ 3 cm, and they examine men of ≥65 years. Recently, AAA prevalence was evaluated in a Northern Italian population recruiting subjects younger than 65 years, including women [16].

The aims of our study were: (1) to estimate AAA prevalence and risk factors in males and females in a Central Italian population; (2) to define the cost-effectiveness of AAA screening programs.

## 2. Materials and Methods

To estimate the sampling dimension (*n* = 4035) the following parameters were used: sample error E = 1.99 and 1-alpha = 0.95.

Our study was a prospective study conducted between 1 January 2015 and 31 December 2019 in the municipality of Teramo (Abruzzo Region, Italy).

Inclusion criteria were: (1) men and women ranging from the age of 65 to 79; (2) who were not previously operated on for AAA.

**Figure 1:**
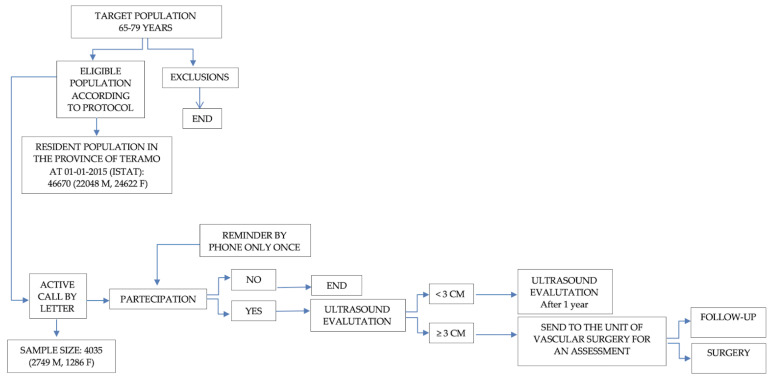
Study flowchart.

Population-group target residents in Teramo, on 1 January 2015, consisted of 466,700 citizens, of which there were 22,048 males and 24,622 females. The citizen was contacted by a letter explaining the scope of the screening and diagnostic test involved; it was followed up by a telephone call a few days before the appointment (Figure 1 flow chart). The citizens that accepted signed a consent form and were asked to fill in a questionnaire that contained socio-demographic and anamnestic variables. The coverage of screening was 57%.

The ultrasound was performed by means of Acuson Sequoia 512 Simens with a Convex probe. The anterior posterior of the infra-renal aorta was evaluated.

In our study, the association between the presence of aortic aneurysm and anamnestic determinants were evaluated by Chi-square test or Fisher’s exact test, when appropriate. Z test for the equality of two proportions was used to verify the differences between gender in AAA diagnosed. The quantitative data were described as a mean ± standard deviation (SD). The odds ratio values (ORs) were used to evaluate the risk of AAA and the determinants taken into consideration.

All analyses were conducted using the software SAS/STAT.

Finally, we estimated the direct costs derived from aneurysmectomy (surgical repair or endovascular aneurysm repair—EVAR) performed in an emergency at Mazzini Hospital in the ordinary regime. The costs were taken from the tariff set by the Abruzzo region [21].

## 3. Results

In the study period, 2749 males and 1286 females were contacted; 2301 (57%) accepted, of which 1529 were males (55.6%, mean age 73.6 ± 4.0) and 772 were females (60%, mean age 74.3 ± 4.1). A total of 62 AAA (2.7%, mean age 73.8 ± 4.0) were diagnosed, of which 57 were in men (3.7%, mean age 73.6 ± 4.0) and 5 were in women (0.7%, mean age 74.3 ± 4.1). All responders were retirees.

**Figure 2:**
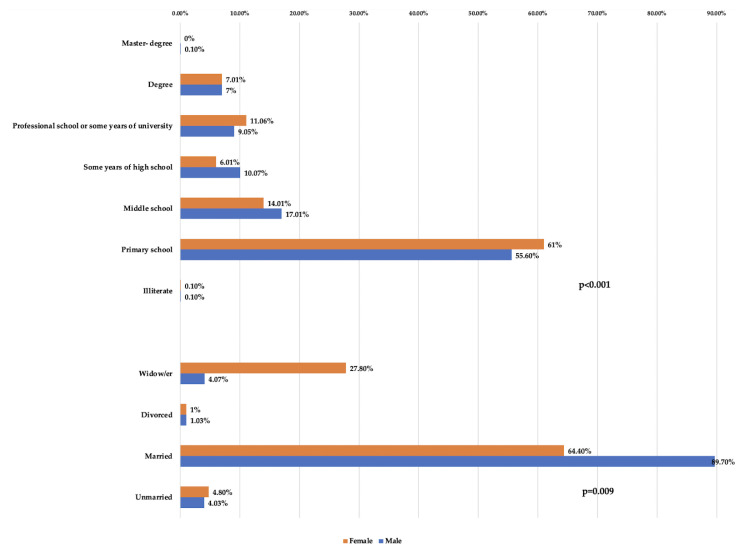
Distribution of socio-demographic variables.

**Figure 3:**
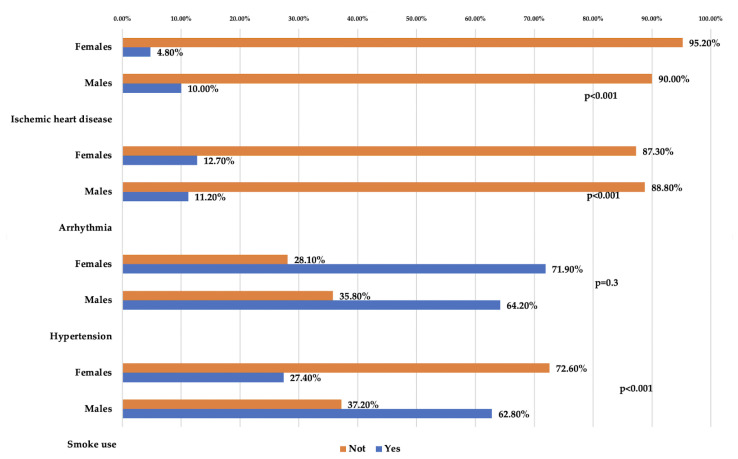
Distribution of comorbidity according to gender.

The socio-demographic and comorbidity of responders are summarized in Table 1 and Table 2 and Figure 2 and Figure 3. Concerning the marital status and education variables, the percentage of primary school and widows was higher in females than in males, *p* < 0.000 and *p* = 0.009, respectively. Smoke use, hypertension, and ischemic heart disease were more frequent in males than females, respectively: 62.8%, 64.2%, and 10.0% (*p* < 0.000). No statistically significant difference for arrhythmia between gender was found.

The associations among AAA ≥ 3 cm and the determinants taken into account are reported in Table 3.

It is important to underline that male gender and smoke use are more important risk factors for AAA ≥ 3 cm, respectively: OR = 5.94 (2.37–14.99 *p* < 0.001) and OR = 5.21 (2.63–10.30, *p* < 0.000).

A significant increase in OR was noted for AAA ≥ 3 cm and cardiac arrhythmia and ischemic heart disease, respectively: OR = 2.81 (1.53–5.15, *p* < 0.000) and OR = 2.76 (1.40–5.43, *p* = 0.006).

Table 4 shows the surgical interventions for AAA in an emergency due to rupture and planned. In the year preceding the activation of the screening, a total of 20 emergency interventions for aneurysm rupture were observed, compared to 16 scheduled interventions. Starting from the following year, there were 6 emergency interventions against 21 in election, a trend confirmed in the following years of screening (Table 4).

Regarding cost analysis, it appears that screening has contributed to the reduction in costs related to urgency. In fact, the synthetic indicator given by the ratio between the DRGs (disease related group) relating to the emergency and those of the elective activity went from 1.69 in the year prior to the activation of the screening to a median of 0.39 for the five-year period of activation of the screening.

## 4. Discussion

The importance of screening campaigns is well known to diagnose a disease early, before the onset of symptoms, and to promptly initiate therapeutic treatment. An early treatment, in fact, has advantages over a late treatment, in terms of recovery, survival from the disease, and simpler treatments. In Italy and Europe, various screening campaigns have been active for many years against breast cancer [22], colorectal cancer [23], and cervical cancer [24]. The Italian National Health System guarantees uniform access throughout the territory to these programs [22].

In the last decade in some European countries [18], but also in other countries [13], abdominal aortic screening (AAS) is emerging as a potential prevention campaign; although, most of the initiatives are local and spontaneous. On the other hand, in the UK, AAS is structured as a population-based screening [25,26].

It has been shown that the risk of rupture of the aneurysm in males is directly correlated with the diameter, so early diagnosis can become a lifesaver [18].

In our population-based study, the prevalence was 2.7%, similar to that reported in the literature [27]. The stratification by gender shows values higher in men (3.8%), with a risk equal to 5.94, than in women (0.65%).

Another interesting aspect that confirms the literature was the higher prevalence of AAA in smokers (4.67%) compared to non-smokers (0.89%). Smoke use is an important risk factor determining an OR = 5.21. A similar result was found by Ahmed et al. [13]. The mechanisms by which smoking predisposes to a greater tendency to aneurysm are not fully clarified. A recent paper from Carnevale et al. underlines the importance of addressing screening campaigns in smokers under 65 years and in non-smoking men over 70 [26]. Sweeting et al. [27] showed that the annual growth of an aneurysm in the smoker is 0.35 mm/year higher than that found in the general population, 2.21 mm/year. This increase is probably due to many factors, such as inflammatory structure, metabolic causes, and others [28]. Recent work on the animal model has shown that smoking contributes to tissue inflammation and apoptosis of vascular smooth muscle cells [29]. The same inflammatory mechanism would be contributing to ischemic heart disease [30]. This evidence could support the increased risk of AAA in smokers and those with a history of ischemic heart disease. Regarding the latter, the results of our study show that AAA is associated with ischemic heart disease (*p* = 0.006) with an OR = 2.76.

Therefore, identifying risk factors becomes crucial when it comes to clinical practice. Especially in light of the limited economic resources that, as Cochrane stated, should be used for health interventions, the effectiveness of which has been proven [31].

**Figure 4:**
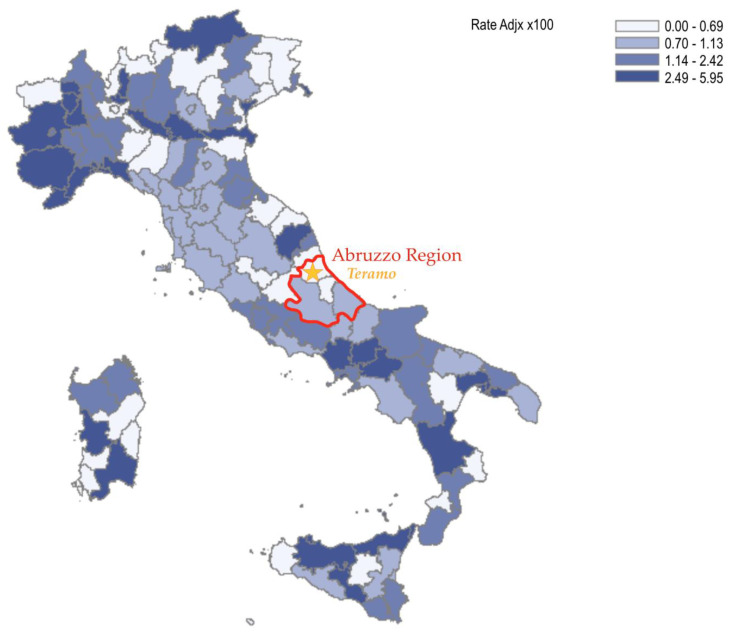
National and Regional death rate for non-ruptured AAA. The color gradient showed the incidence rate of AAA in each Italian province.

Giardinia et al. [32], in a study conducted on the Italian population, showed that abdominal aortic screening is cost-effective. This evidence also results from a recent meta-analysis by Ying et al. [33], which showed that screening reduces mortality. The mortality found for AAA in our area where screening was activated was 1.025%; this result is lower than the national Figure 1.7% (Figure 4) [34].

A similar reduction in AAA mortality is present in the study by Ashton et al. [35] and confirmed by several meta-analyses. [11,33].

The results of our work confirm that the screening activated in our territory has led to a reduction in the expenditure for AAA emergency interventions, having increased the planned interventions (Table 3). It seems important to underline that simulation models highlight how the cost-benefit of this initiative is maintained even if applied in different national contexts [32,36].

This must be a warning for local stakeholders, especially in the post-pandemic period, to strengthen prevention. In particular, with regard to the AAA, an effort in terms of resource allocation could be addressed primarily to the screening of smoking patients and patients with ischemic heart disease (the latter easily identifiable on the basis of hospital records or pharmacological prescriptions). Wild et al. [37] showed that aortic dilations between 25 mm and 29 mm, in 8.3% of cases, can evolve over 13.4 years to an aneurysm.

A decisive role could be played by family doctors in identifying high-risk patients, promoting their control through ultrasound screening, and making people aware of taking action on modifiable risk factors relating to lifestyles [38], with particular reference to smoking.

## 5. Conclusions

Screening for an abdominal aortic aneurysm is a useful investigation to reduce the specific mortality of this pathology. Furthermore, considering the high prevalence of AAA, the often-silent clinic and the high mortality related to its more fearful complication, rupture, a key role in early diagnosis, or at least in diagnostic suspicion, is played by the general practitioner (GP). In fact, GPs are well aware of their patients’ lifestyles, such as the use of alcohol and smoking, which represent important risk factors. The GP, as part of the assessment of the patient, is, therefore, the first line in acknowledging or suspecting a potentially fatal condition. In addition, the ultrasound examination is suitable, due to its non-invasive characteristics and easy applicability, as a first exam level for the identification of the AAA, which can also be implemented in the suitably trained GP’s medical office.

## Figures and Tables

**Table 1:** Socio-demographic variables.

Variables	Responders	Gender		
		No. (%) Males	No. (%) Females	*p*-Values
**Marital status**				<0.000 Χ^2^ = 263.4
Unmarried	92	59 (4.3)	33 (4.8)	
Married	1684	1241 (89.7)	443 (64.4)	
Divorced	27	20 (1.3)	7 (1.0)	
Widow/er	268	63 (4.7)	205 (27.8)	
Total responders	2071	1383	688	
**Education**				0.009 Χ^2^ = 18.7
Illiterate	3	2 (0.1)	1 (0.1)	
Primary school	1188	769 (55.6)	419 (61.0)	
Middle school	333	236 (17.1)	97 (14.1)	
Some years of high school	190	148 (10.7)	42 (6.1)	
Professional school or some years of university	211	131 (9.5)	80 (11.6)	
Degree	146	97 (7.0)	49 (7.1)	
Master’s degree	1	1 (0.1)	0 (0.0)	
Total responders	2072	1384	688	

**Table 2:** Comorbidity distribution according to gender.

Gender	Responders	No. (%) Yes	No. (%) Not	*p*-Values
**Smoke Use**				
Males	1523	956 (62.8)	567 (37.2)	<0.000 Χ^2^ = 256.7
Females	771	211 (27.4)	560 (72.6)	
Total responders	2294	1167	1127	
**Hypertension**				
		No. (%) Yes	No. (%) Not	<0.000 Χ^2^ = 12.3
Males	1380	886 (64.2)	494 (35.8)	
Females	684	492 (71.9)	192 (28.1)	
Total responders	2064	1378	686	
**Arrhythmia**				0.3 Χ^2^ = 1.1
		No. (%) Yes	No. (%) Not	
Males	1378	154 (11.2)	1224 (88.8)	
Females	684	87 (12.7)	597 (87.3)	
Total responders	2062	241	1821	
**Ischemic heart disease**				<0.000 Χ^2^ = 16.2
		No. (%) Yes	No. (%) Not	
Males	1379	138 (10.0)	1241 (90.0)	
Females	684	33 (4.8)	651 (95.2)	
Total responders	2063	171	1892	

**Table 3:** Association and risk factors of AAA according to variables considered.

**Gender and Abdominal Aortic Diameter**				
	**Diameter**			
Gender	No. (%) < 3 cm	No. (%) ≥ 3 cm	Total	Χ^2^ = 18.6 *p* ≤ 0.000 OR: 5.94 (2.37–14.88)
Males	1472 (96.3)	57 (3.7)	1529	
Females	767 (99.4)	5 (0.6)	772	
Total	2239	62	2301	
**Current smokers or former smokers (** **≥** **10 cigarettes/day)**				
	Diameter			
Smoke use	No. (%) < 3 cm	No. (%) ≥ 3 cm	Total	Χ^2^ = 27.8 *p* ≤ 0.000 OR: 5.21 (2.63–10.30)
Yes	1115 (95.5)	52 (4.5)	1167	
Not	1117 (99.1)	10 (0.9)	1127	
Total responders	2232	62	2294	
**Cardiac arrhythmia and abdominal aortic diameter**				
	Diameter			
Arhythmia	No. (%) < 3 cm	No. (%) ≥ 3 cm	Total	Χ^2^ = 12.8 *p* ≤ 0.000 OR: 2.81 (1.53–5.15)
Yes	226 (93.8)	15 (6.2)	241	
Not	1779 (99.1)	42 (2.3)	1821	
Total responders	2005	57	2062	
**Ischemic heart disease and abdominal aortic diameter**				
	Diameter			
Ischemic heart disease	No. (%) < 3 cm	No. (%) ≥ 3 cm	Total	Χ^2^ = 27.8 *p* = 0.006 OR: 2.76 (1.40–5.43)
Yes	160 (96.3)	11 (6.4)	171	
Not	1846 (97.6)	46 (2.4)	1892	
Total responders	2006	57	2063	
**Hypertension and abdominal aortic diameter**				
	Diameter			
Hypertension	No. (%) < 3 cm	No. (%) ≥ 3 cm	Total	Χ^2^ = 2.1 *p* = 0.149 OR: 0.68 (0.40–1.15)
Yes	1345 (97.6)	33 (2.4)	1378	
Not	662 (96.5)	24 (3.5)	686	
Total responders	2007	57		

**Table 4:** Screening according to surgery and costs.

	Emergency				Planned				Costs of Emergency Euro	Costs Planned Euro	Screening Coasts/Year Euro	Ratio Emergency/ Planned
	Evar	Open	TOT	Costs Drg 110 Euro	Evar	Open	Tot	Costs DRG 111 Euro				
2014	4	16	20	13,874.36			16	10,253.09	277,487.20	164,049.44	Inactive screening	1.69
2015	2	4	6				21		83,246.16	215,314.89	14,726.40	0.39
2016	2	8	10				20		138,743.60	205,061.80	14,726.40	0.68
2017	1	0	1		12	7	19		13,874.36	194,808.71	14,726.40	0.07
2018	0	0	0		8	3	11		-	112,783.99	14,726.40	0.00
2019	2	0	2		6	1	7		27,748.72	71,771.63	14,726.40	0.39
									541,100.04	963,790.46	73,632.00	0.56

DRG: DIAGNOSIS RELATED GROUPS. DRG 110: major intervention on cardiovascular system with complications EUR 13,874.36. DRG 111: major intervention on cardiovascular system without complications EUR 10,253.09.

## Data Availability

No data supporting.

## Abbreviations

AAA	Abdominal aortic aneurysm
DRG	disease related group
EVAR	Endovascular aneurysms repair
